# Prevalence of Post-traumatic Stress Disorder After Flood: A Systematic Review and Meta-Analysis

**DOI:** 10.3389/fpsyt.2022.890671

**Published:** 2022-06-23

**Authors:** Mohamad Golitaleb, Elaheh Mazaheri, Mahtab Bonyadi, Ali Sahebi

**Affiliations:** ^1^Department of Nursing, School of Nursing, Arak University of Medical Sciences, Arak, Iran; ^2^Health Information Technology Research Center, Student Research Committee, Isfahan University of Medical Sciences, Isfahan, Iran; ^3^Non-communicable Diseases Research Center, Ilam University of Medical Sciences, Ilam, Iran

**Keywords:** Post-traumatic stress disorder, PTSD, mental disorders, natural disasters, flood

## Abstract

**Introduction:**

Flood as the most common kind of the natural disasters has unpleased short, medium, and long-term consequences on the victims’ welfare, relationships, and physical and mental health. One of the most common mental health disorders in these victims is Post-traumatic stress disorder (PTSD). The aim of this study is to investigate the prevalence of PTSD on the flood victims.

**Methods:**

Data resources including PubMed, Scopus, Web of Science, Science Direct, Embase, Google Scholar, conference and congress papers, key journals, the reference list of selected articles as well as systematic reviews were searched to identify studies that reported the prevalence of PTSD in flood victims. Random Effect Model was used to perform meta-analysis of the studies. Cochran test and I^2^ indicator were used to explore heterogeneity between the studies. Publication bias of the study was evaluated using Begg’test. Data were analyzed by STATA (version 14) software.

**Results:**

After a comprehensive search, 515 papers were extracted. After eliminating duplicates and final screening, 23 studies were selected and entered the meta-analysis phase after qualitative evaluation. The results showed that the prevalence of PTSD in flood victims is 29.48% (95% CI: 18.64–40.31, I^2^ = 99.3%, *p*-value < 0.001).

**Conclusion:**

The results of the present study showed that the prevalence of PTSD is relatively high in the flood victims. So, it is necessary to take preventive, supportive, therapeutic and effective actions for them.

## Introduction

Flood is the most common kind of natural disaster and climate changes have increased its incidence (1). It also has obvious effects on the individuals’ life and in addition to economic damages, it has unpleased short, medium and long term consequences on the victims’ welfare, relationships and physical and mental health (1, 2). Disasters can have severe mental health consequences which are the result of encountering to the potentially traumatic events and tenacious stressors (3, 4). Floods make some changes in the people routine lives, so that their quality of life would be affected in different manners (5). Floods are associated with an increased prevalence of mental disorders such as Post-traumatic stress disorder (PTSD), anxiety and depression in industrialized and non-industrialized countries (6). On the other hand, the studies have shown that PTSD can lead to other mental health problems such as depression, anxiety or substance abuse disorders (1). PTSD occurs as a delayed reaction to very threatening or catastrophic conditions in the short or long term (2). It disturbs the individual’s daily life function and is associated with decreased health function and increased physical and mental illness (7). The results of a study in Indonesia showed that 52% of flood victims had experienced PTSD and 98.3% of them experienced it again (8). A study in China had reported the prevalence of PTSD 9.2% among the floods victims (9). According to the results of a review study, the prevalence of PTSD was 28.44% among the earthquake victims (10). In a systematic review and meta-analysis study was conducted in 2015, the incidence of PTSD after the flood was 15.74% (11). The rate of the psychiatric illnesses in the victims have been varied based on some factors such as the vulnerable population, severity and type of the flood event as well as the social support of the country (12). Since the flood has various physical and mental consequences in the long and short term, no timely detection and diagnose of long-term psychological consequences can be followed by unpleasant circumstances that may disrupt the individual’s daily life. Considering that until 2015, the rate of PTSD after the flood has been reported using a systematic review and meta-analysis study and Several studies have been carried out on the prevalence of PTSD after the flood, but no comprehensive study has been done since 2015 on the overall prevalence of PTSD. Therefore, the present study was conducted to assess the prevalence of PTSD in flood survivors by a systematic review and meta-analysis. The results of the study can be used for planning and policy-making for the follow-up actions for the flood survivors.

## Materials and Methods

In order to conduct the present review study the PRISMA (Preferred reporting items for systematic reviews and meta-analyses) guideline was used (13). The review protocol was registered in the International Prospective Register of Systematic Reviews (PROSPERO) under the code of CRD42021281715.

### Search Strategy

The English information resources including PubMed, Scopus, Web of Science, Science Direct, Google Scholar as well as Embase were searched by English keywords to retrieve the related studies. Also, other data resources, including conference and congress papers, key journals, the reference list of selected articles and systematic reviews were used. To formulate the search strategy in databases, keywords, tag field and operators were used. Searche English language were carried out from the beginning of 2015 to 31 June 2021. The search strategies for the types of databases were provided in Supplementary Appendix 1.

### Inclusion Criteria

The studies that had reported the prevalent of PTSD among the flood survivors from the beginning of 2015 to 31.05.2021 were included in this review.

### Exclusion Criteria

(1) Review, qualitative study and intervention studies, (2) Studies other than English language were the exclusion criteria of the study.

### Selection of the Studies

In order to manage the references, all the initial identified studies were entered into the Endnote software (version 7) and after deleting duplicates, titles and abstracts of 478 studies were screened. In the next step, two researchers (AS and MG) independently studied 50 possible related studies by details and finally 23 papers were selected. Any disagreement between them was resolved by a third person.

### Qualitative Evaluation and Data Extraction

In order to evaluate the quality of the selected studies, two researchers (AS and MG) independently used Appraisal tool for Cross-Sectional Studies (AXIS) Tool (14). The tool has the score between 0 and 20. Based on the AXIS tool, higher scores indicate higher quality studies. Any disagreement between two evaluators was resolved by a third individual. Data extraction of all included studies was done by two researchers (AS and MG) independently by using a pre-prepared checklist. This checklist included these items: (a) the name of the first author, (b) the place, (c) the year and (d) the tool of the study, (e) sample size, the number of men and women, as well as the prevalence of PTSD. Any disagreement between two researchers was resolved by a third individual.

### Statistical Analysis

Given that the prevalence of PTSD and the sample size were extracted in each study, a binomial distribution was used to calculate the variance of each study. The weighted average was used to combine the prevalence of different studies. Each study was weighted according to its inverse variance. Due to high heterogeneity, the simple random effects model was used for meta-analysis. The heterogeneity between the studies was calculated using the Cochran test and I^2^ indicator. The scores less than 25, 25–50, 50–75, and more than 75% indicates, no; moderate, high as well as very high heterogeneity, respectively (15). *P* < 0.05 was considered significant in the Cochrane test. Subgroup analysis was used to explore the source of heterogeneity. Publication bias of the study was assessed using Begg’ test. The significance level in the Begg’ test is 0.05 (16). Sensitivity analysis was performed to identify the effect of any single study on the overall prevalence. To investigate the relationship between the year of the study and the prevalence of PTSD, Meta-regression was used and the data were analyzed by STATA software (Version 14).

## Results

**FIGURE 1:**
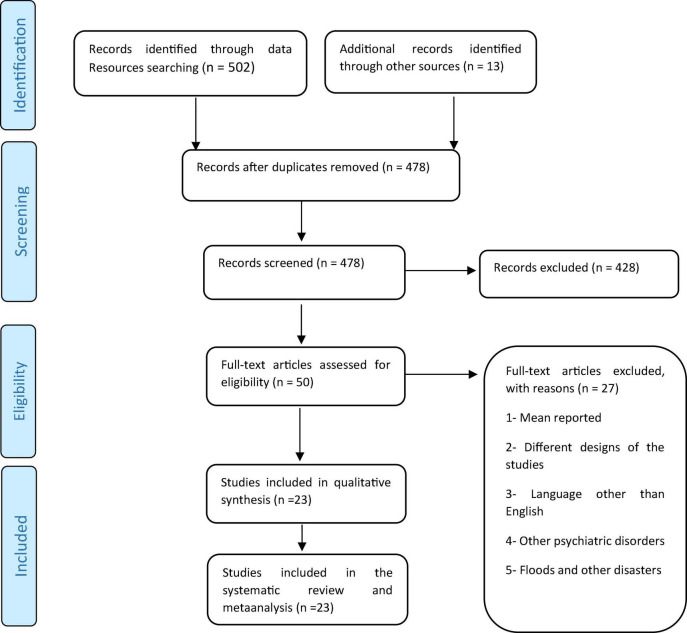
Flowchart of the study and selection of studies based on PRISMA steps.

**FIGURE 2:**
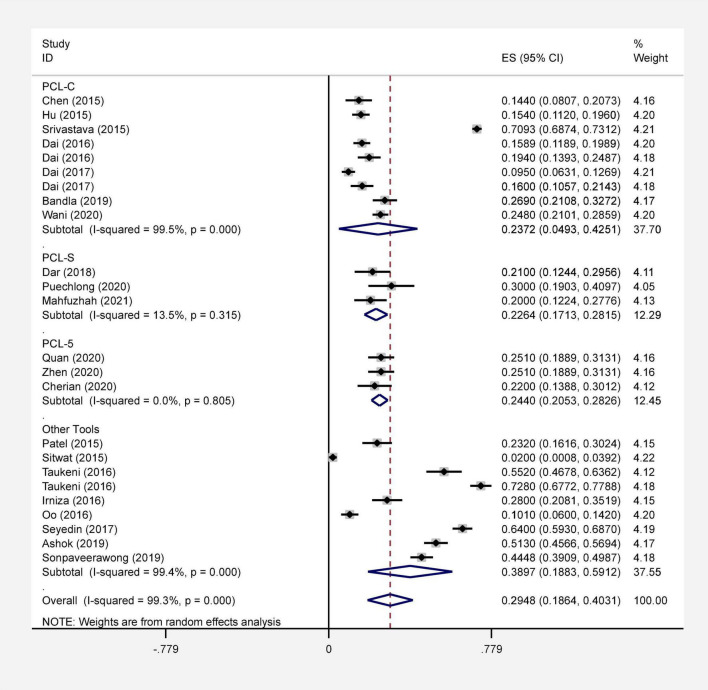
Forest plot of the prevalence of PTSD after the flood in general and separately with 95% confidence interval.

In the present review, 515 articles were retrieved by the initial search and after deleting the duplicates, 478 papers were screened. After the final screening, 23 articles were selected and assessed and they all entered the meta-analysis (Figure 1). All studies included were conducted between 2015 and 2021. Based on the AXIS tool, the quality score of the included studies varied from 12 to 19. The methodology of all selected studies was cross-sectional (Table 1). In this study, PTSD of the flood victims was reported 29.48% (95% CI: 18.64–40.31, I^2^ = 99.3%, *p*-value < 0.001) (Figure 2). Indicator I^2^ showed that the heterogeneity between the studies was very high.

**TABLE 1:** The characteristics of the selected studies in meta-analysis.

References	Tool**	Country	Sample size	Male	Female	Prevalence of PTSD
Bandla et al. (17)	PCL-C	India	223	107	116	26.9%
Chen et al. (18)	PCL-C	China	118	59	59	14.4%
Dai et al. (19)	PCL-C	China	321	173	148	15.89%
Dai et al. (20)	PCL-C	China	325	153	172	9.5%
Dai et al. (21)	PCL-C	China	175	81	94	16%
Dai et al. (22)	PCL-C	China	201	111	90	19.4%
Hu et al. (23)	PCL-C	China	284	153	131	15.4%
Irniza et al. (24)	TSQ	Malaysia	150	61	89	28%
Puechlong et al. (25)	PCL - S	France	67	32	35	30%
Quan et al. (26)	PCL-5	China	187	94	84 (9*)	25.1%
Sitwat et al. (27)	SCID-IV	Pakistan	205	–	205	2%
Sonpaveerawong et al. (28)	GHQ- 12- Plus-R	Thailand	326	177	149	44.48%
Taukeni et al.(29)	CTSQ	Southern Africa	134	44	90	55.2%
Taukeni et al. (29)	CTSQ	Southern Africa	295	114	181	72.8%
Zhen et al. (30)	PCL-5	China	187	–	–	25.1%
Oo et al. (31)	Researcher made	Malaysia	208	98	110	10.1%
Mahfuzhah et al. (32)	PCL - S	Indonesia	102	51	51	20%
Srivastava et al. (33)	PCL-C	India	1651	739	912	70.93%
Seyedin et al. (34)	PTSS-10	Iran	400	238	162	64%
Ashok et al. (35)	IES-R	India	302	120	182	51.3%
Wani (36)	PCL-C	India	500	171	329	24.8%
Cherian et al. (37)	PCL-5	India	100	29	71	22%
Patel et al. (38)	IES-R	India	138	–	–	23.2%
Dar et al. (39)	PCL - S	India	87	39	48	21%

*Unknown gender.

**PCL - S, post-traumatic stress disorder checklist – Specific; IES – R, impact of event scale – revised; PCL – 5, post-traumatic stress disorder checklist for DSM-5; PCL-C, post-traumatic stress disorder checklist - civilian version; PTSS-10, post-traumatic symptom scale-10; CTSQ, child trauma screening questionnaire; GHQ-12-Plus-R, general health questionnaire twelve plus R; TSQ, trauma screening questionnaire; SCID – IV, structured clinical interview for DSM – IV disorders.

### Subgroup Analysis

The research team performed a subgroup analysis using the type of tool used to assess the prevalence of PTSD. The prevalence of PTSD in flood victims based on PCL-C, PCL-S, PCL-5, and other tools were reported 23.27% (95% CI: 4.93–42.51, I^2^ = 99.5%, *p*-value < 0.001), 22.64% (95% CI: 17.13–28.15, I^2^ = 13.5%, *p*-value 0.315), 24.40% (95% CI: 20.53–28.26, I^2^ = 0.0%, *p*-value 0.805) and 38.97% (95% CI: 18.83–59, I^2^ = 99.4%, *p*-value < 0.001), respectively (Figure 2). Indicator I2 showed that the heterogeneity between the studies was very high in PCL – C (I^2^ = 99.5%) and no heterogeneity in PCL – 5 (I^2^ = 0.0%).

### Meta-Regression, Publication Bias, and Sensitivity Analysis

**FIGURE 3:**
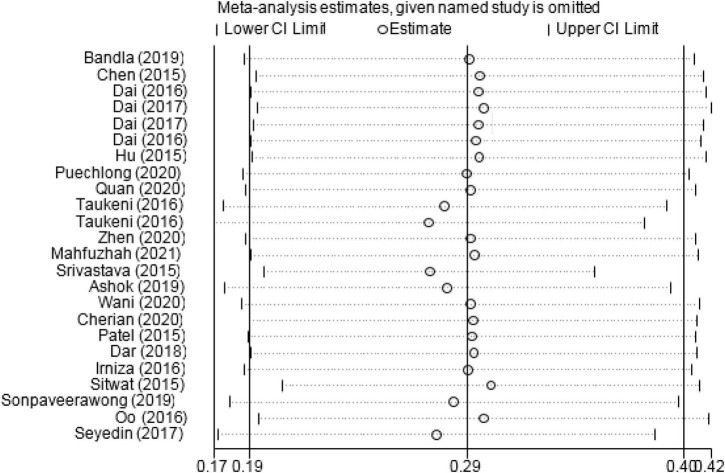
Sensitivity analysis for the prevalence of PTSD.

Based on the Begg test results, the publication bias of the subgroups analysis was not significant (PCL – C: *P*-value = 0.532, PCL – S: *P*-value = 0.117, PCL - 5: *P*-value = 0.297 and Other Tools: *P*-value = 0.677). The result of sensitivity analysis showed that the prevalence of PTSD did not change after the remove of any single study (Figure 3).

## Discussion

This review study assessed the prevalence of PTSD of the flood victims. In total, 23 studies were evaluated by meta-analysis. The results of the study showed that the prevalence of PTSD after the flood was 29.48% and the heterogeneity between the studies was 99.3%. Another meta-analysis study was conducted in 2015 and the results of the assessments on 14 studied papers showed that the incidence of PTSD after the flood was 15.74%. The degree of heterogeneity between the studies using the I^2^ Index was reported 98.3% (11). The results of another meta-analysis study showed that the prevalence of PTSD after the earthquakes and floods in the children and teenager in the first, second, third and fourth 6-month period was 19.2, 30, 24.4 as well as 20.4%, respectively (40). The results of another review study showed the prevalence of PTSD among Health Care Workers during the COVID-19 was 13.52% (41). By comparing the results of the present study with the previous studies, it can be concluded that the prevalence of PTSD after the flood is high like other natural disasters and all age groups are at risk of mental disorders after the flood, especially PTSD. Therefore, it is suggested to the support organizations to screen the flood survivors precisely in different periods. Parallel to the previous researches, the results of this study also showed very high heterogeneity among the studies, which can be resulted from the different sample sizes and various tools used in the studies. Given that each of the tools used in the studies has a different cut-off point, so it can increase the heterogeneity between the studies.

The results of this study showed that the most of the researches have been conducted in Asia and also based on the results of subgroup analysis, they were carried out in India and China with (34%) and (17%), respectively. Therefore, it can be concluded that the prevalence of PTSD in Asia is higher than in any other regions. The results of another study showed that the highest prevalence of PTSD in Asia is 57.3% and it was associated with the natural disasters (42). On the other hand, according to the epidemiological studies of the disasters, Asia has the highest rate of the natural disasters and the frequency and severity of the disasters is increasing (43). Based on the results of the studies, the experience of traumatic life significantly increases the probability of mental health disorders such as PTSD and these traumatic experiences have a greater impact on the mental health of the women than men (44). So, regarding to the results of the previous study and the present study, it can be concluded that the frequency of natural disasters in Asia and re-experience of the traumatic event by the individuals can increase the incidence of PTSD in these areas.

Gender is another factor that can affect the prevalence of PTSD in the victims of the floods and other disasters. According to the studies, PTSD was higher in female survivors after the earthquake than male (45). Therefore, the women are at more risk to suffer from PTSD after the disasters. This finding is in accordance with the previous studies (46–49). The results of other studies have also shown that the people who have lost their properties after the floods or were not supported by their families, like widowed or divorced women, have suffered from more stress and their scores of PTSD were more (50, 51). Other studies have shown that risk factors such as gender, socioeconomic status, education, and symptoms of previous mental illness are associated with increased vulnerability of survivors’ mental health in natural disasters (52). Therefore, it is suggested that communities and support organizations use various strategies such as increasing community resilience and awareness, as well as improving access to the health and treatment cares to raise post-disaster adaptation.

The results of the present study showed that the prevalence of PTSD has a relatively decreasing trend in the flood survivors over time. The results of another review study that looked at the prevalence of PTSD in earthquake survivors showed that the prevalence of PTSD decreased over time (53, 54). Therefore, it can be concluded that the prevalence of PTSD following natural disasters has been declining. Nowadays, the factors like increasing public awareness and resilience, screening, as well as easy access to public health facilities can justify the decreasing trend of the prevalence of mental disorders, especially, PTSD in natural disasters survivors.

### Study Limitations

Although disasters occur around the world and are studied globally, there are intercultural limitations in the use of PTSD assessment tools that have been originally designed and approved in developed countries. It is necessary to be more careful about the inferences that can be drawn from studies using these tools. Another limitation of this study was that most studies were performed in Asia, so the generalization of the results of this study to the whole world may be limited, so it is recommended that other studies be performed to assess PTSD in other parts of the world.

## Conclusion

The results of the present systematic review and meta-analysis study showed that the prevalence of PTSD in the flood victims is relatively high. Therefore, it can affect negatively on their habits, behaviors, interests and lifestyle and create more mental and physical disorders. So, it is necessary to take preventive, supportive, therapeutic and effective actions. On the other hand, the findings of this study can be used as a basis for planning and policy-making in the follow-up of flood survivors. As due to the various problems, floods occur frequently in the developing countries, the suffered populations of these countries need more attention and care. To speed up the curing process and using the appropriate and timely treatment along with other therapeutic actions, it is required to prevent or at least reduce the psychological consequences of the flood.

## Data Availability Statement

The original contributions presented in this study are included in the article/Supplementary Material, further inquiries can be directed to the corresponding author.

## Author Contributions

AS, MB, EM, and MG designed the review, developed the inclusion criteria, screened titles and abstracts, appraised the quality of included manuscript, drafted the manuscript, and reviewed the study protocol and inclusion criteria and provided substantial input to the manuscript. AS and MG read and screened articles for inclusion. All authors critically reviewed drafts and approved the final manuscript.

## Conflict of Interest

The authors declare that the research was conducted in the absence of any commercial or financial relationships that could be construed as a potential conflict of interest.

## Publisher’s Note

All claims expressed in this article are solely those of the authors and do not necessarily represent those of their affiliated organizations, or those of the publisher, the editors and the reviewers. Any product that may be evaluated in this article, or claim that may be made by its manufacturer, is not guaranteed or endorsed by the publisher.
