# Supplementary material for: Prevalence of Post-traumatic Stress Disorder After Flood: A Systematic Review and Meta-Analysis
Source: Front Psychiatry. 2022 Jun 23;13:890671. doi: 10.3389/fpsyt.2022.890671 (PMC9259936; doi:10.3389/fpsyt.2022.890671)
Supplement: Supplementary file 1 [file Data_Sheet_1.docx]

Appendix 1: Search strategy for the types of databases

| Data base | Search strategy |
| --- | --- |
| PubMed | (“Catastrophic Flooding*” OR Flood) AND (“posttraumatic stress disorder” OR “posttraumatic neuroses” OR “Post Traumatic Stress Disorder*” OR “chronic post-traumatic stress disorder” OR “delayed-onset post-traumatic stress disorder” OR “acute post-traumatic stress disorder” OR PTSD) |
| Scopus | ((ALL(“Catastrophic Flooding*”) OR ALL(Flood)) AND (ALL(“posttraumatic stress disorder”) OR ALL(“posttraumatic neuroses”) OR ALL(“Post Traumatic Stress Disorder*”) OR ALL(“chronic post-traumatic stress disorder”) OR ALL(“delayed-onset post-traumatic stress disorder”) OR ALL(“acute post-traumatic stress disorder”) OR ALL(PTSD))) |
| Web of science | ((TS=(“Catastrophic Flooding*”) OR TS= (Flood)) AND (TS= (“posttraumatic stress disorder”) OR TS= (“posttraumatic neuroses”) OR TS= (“Post Traumatic Stress Disorder*”) OR TS= (“chronic post-traumatic stress disorder”) OR TS= (“delayed-onset post-traumatic stress disorder”) OR TS= (“acute post-traumatic stress disorder”) OR TS= (PTSD))) |
